# Hippocampal sparing in whole-brain radiotherapy for brain metastases: controversy, technology and the future

**DOI:** 10.3389/fonc.2024.1342669

**Published:** 2024-01-24

**Authors:** Rui Liu, GuanZhong Gong, KangNing Meng, ShanShan Du, Yong Yin

**Affiliations:** ^1^ Department of Graduate, Shandong First Medical University, Shandong Academy of Medical Sciences, Jinan, China; ^2^ Department of Radiation Oncology Physics and Technology, Shandong Cancer Hospital and Institute, Shandong First Medical University and Shandong Academy of Medical Sciences, Jinan, China

**Keywords:** hippocampus avoidance, brain metastases, whole brain radiotherapy, new radiotherapy techniques, contour, dose limits

## Abstract

Whole-brain radiotherapy (WBRT) plays an irreplaceable role in the treatment of brain metastases (BMs), but cognitive decline after WBRT seriously affects patients’ quality of life. The development of cognitive dysfunction is closely related to hippocampal injury, but standardized criteria for predicting hippocampal injury and dose limits for hippocampal protection have not yet been developed. This review systematically reviews the clinical efficacy of hippocampal avoidance - WBRT (HA-WBRT), the controversy over dose limits, common methods and characteristics of hippocampal imaging and segmentation, differences in hippocampal protection by common radiotherapy (RT) techniques, and the application of artificial intelligence (AI) and radiomic techniques for hippocampal protection. In the future, the application of new techniques and methods can improve the consistency of hippocampal dose limit determination and the prediction of the occurrence of cognitive dysfunction in WBRT patients, avoiding the occurrence of cognitive dysfunction in patients and thus benefiting more patients with BMs.

## Introduction

Brain metastases (BMs) are the most common central nervous system (CNS) malignancies and may occur at a rate more than 10 times greater than that of primary malignant tumors of the brain (1). Commonly used treatments for BMs include surgery, radiotherapy (RT), and systemic therapies (immunotherapy, targeted drugs). Since 60% of patients have multiple lesions, 5% of them have concurrent meningeal metastases and significant neurological symptoms (2). Therefore, whole-brain RT (WBRT) plays an irreplaceable role in the treatment of BMs. WBRT increases the median survival time to 3-6 months and completely relieves headache and intracranial hypertension in more than 50% of patients, making it the treatment of choice for patients with multiple lesions and significant symptoms (3). However, even though modern local RT techniques are improving tumor control, cognitive dysfunction caused by WBRT has become a non-negligible problem in the current clinical application of WBRT. It is especially important for patients who can survive for a long time. A significant dose−effect relationship exists between cognitive decline after RT and the dose received in the hippocampus (4). Hippocampal avoidance-WBRT (HA-WBRT) has become the routine technique of choice for treating BMs during WBRT. Several scholars have reviewed the HA-WBRT protocol and summarized the clinical efficacy, technical feasibility, potential neurotoxicity, hippocampal dose limits, and characteristics of different RT techniques implemented in HA-WBRT (5–8).

This review addresses the current problems faced by HA-WBRT in terms of differences in clinical efficacy and varying standards of hippocampal dose limits and summarizes the imaging characteristics of the hippocampus on computed tomography (CT) and images and magnetic resonance imaging (MRI). The differences in hippocampal protection induced by commonly used RT techniques include volumetric modulated arc therapy (VMAT), helical Tomotherapy (Tomo), and intensity-modulated proton therapy (IMPT), as well as the application of hippocampal automatic and semi-automatic segmentation methods, these findings provide insight into the direction of the future development of HA-WBRT.

## Controversies regarding HA-WBRT

Although preserving the hippocampus has become the standard protocol for WBRT, some scholars have questioned the efficacy of HA-WBRT, as originally proposed by Gondi et al. (9) Gondi et al. and Westover KD et al. (10) achieved good clinical outcomes in their studies of HA-WBRT, in which 23% and 49.4%, improved cognitive function preservation, respectively. Conversely, the HA-WBRT studies by Beblerbos et al. (11) and Vees et al. (12) did not yield significant clinical results. **Table 1** lists the controversies studied by scholars.

**Table 1:** The controversies of HA-WBRT clinical outcome.

	Study	Neuropsychological test time point	Primary end points	Result
Support	Gondi et al. (2014) (9)	When baseline, 2, 4, and 6 months HVLT-R DR	4 month‘s HVLT-R DR	Cognitive score with HA-WBRT VS WBRT decline:7.0%Vs 30%
	Westover et al. (2020) (10)	When baseline, 3, 6, 9, 12 months HVLT-R	3 month’s HVLT-R	Cognitive score with HSIB-WBRT VS WBRT decline: 10.6% Vs : 60%
Oppose	Beblerbos et al. (2020) (11)	When baseline, 4, 8, 12, 18, 24 months HVLT-R	4 month’s HVLT-R	HA-PCI NCF VS PCI dropped to five points: 28%Vs 29%
	Vees et al. (2020) (12)	When baseline 6 weeks, 6and 12months HVLT-R	NCF decline at 6 months	No NCF decline at 6VS 12 months: 34.2% VS 48.5%

HVLT-R, Hopkins Verbal Learning Test -Revised; HVLT-DR, Hopkins Verbal Learning Test -Delay Revised; PCI, Prophylactic Cranial Irradiation.

## Progress in the study of hippocampal dose limits in WBRT

There are more studies on the dose limits for hippocampal injury, but no uniform conclusion has been reached. **Table 2** lists the studies of several scholars on hippocampal protective dose limits (13–17).

**Table 2:** The dose limitation of hippocampus studied by several scholars.

Study	Prescription dose	Hippocampus dose limitation		
		Left	Right	Bilateral hippocampal
Tsai et al. (2015) (13)	EQD2(25Gy)			EQD-20%<12.60Gy
Goda et al. (2020) (14)	54Gy	Dmean<30Gy		
Pospisic et al. (2016) (15)	30Gy	Dmean<23.7Gy	Dmean<28.8Gy	
Ma et al. (2017) (16)	25Gy			D50%<22.1Gy
Gondi et al. (2011) (17)	EQD2(20-54Gy)			EQD2-40%<7.3Gy

EQD2, Equivalent Doses in 2-Gy Fractions.

One of the primary reasons for the contradictory findings among scholars may be the lack of a precise definition of the hippocampal region and varying criteria for contouring, leading to different actual hippocampal exposure doses and consequently different outcomes. The latest guidelines for assessing hippocampal contours, as defined in the RTOG0933 study, do not include the entire hippocampus but focus on a subregion of granule cells in the dentate gyrus, which is crucial for memory formation and challenging to contour clearly and reliably by imaging (18). The criteria for dose limits based on hippocampal boundaries continue to require discussion. The initial step in RT involves contouring the tumor target area and identifying critical organs such as the hippocampus. This step is pivotal and dictates the success of the entire RT regimen.

## Progress of hippocampal contours

The hippocampus is an elongated structure located deep in the medial temporal lobe that anatomically resembles the hippocampus (19). Its structure is mainly C-shaped at the base of the temporal horn of the lateral ventricle, with a rostral projection approximately 5 cm long (20). Unlike other vulnerable brain structures, such as the brainstem or optic chiasm, the hippocampus is situated within the temporal lobe. It has unique anatomical features characterized by a small size and complex shape. These attributes make accessing hippocampal contours time-consuming and challenging. Furthermore, the hippocampus appears different in CT images and MRI, each playing a distinct role in determining the hippocampal contour.

## Imaging basis for hippocampal contours

### CT

**Figure 1:**
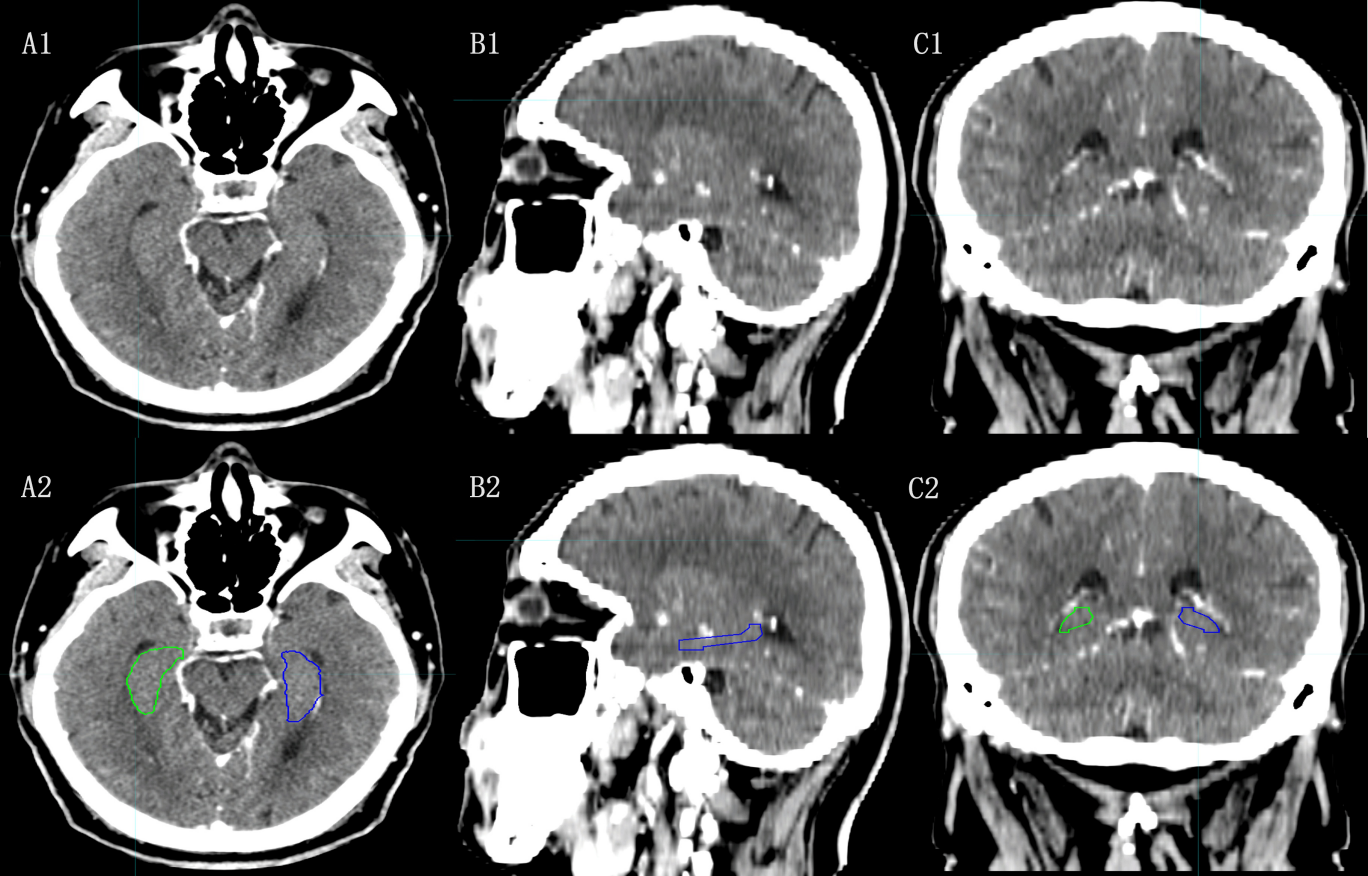
The hippocampal contour based on CT images. [**(A1)**, **(B1)**, **(C1)**:Axial, Sagital and Coronal images of CT; **(A2)**, **(B2)**, **(C2)**: Axial, Sagital, Coronal images of CT with hippocampal contour].

In the HA-WBRT context, identifying the hippocampus is the initial step. However, modern RT techniques rely on contouring target areas using CT scans, and localizing the hippocampus on CT images presents challenges. Nangia et al. (21) attempted to trace the hippocampal edges using CT imaging by adjusting the window width and position in their study of radiation treatment for alveolar carcinoma. Unfortunately, this approach has limitations due to the low tissue resolution of CT scans, which makes it difficult to distinguish gray matter structures within hippocampal boundaries (**Figure 1**).

In the previous HA-WBRT studies, hippocampal identification was accomplished using high-resolution T1 weighted imaging (T1W1) of MR scans (22). Merging CT images and MRI can enhance hippocampal recognition and contouring. However, combining CT and MRI introduces additional errors and distorts the MRI (23).

Porter et al. (24) developed an attention-graded-3D ResNet (AG-3D ResNet) model for automatic segmentation of the hippocampus based on CT scan images. They compared the model with a model built based on MR-CT fusion and images for accuracy and reliability. The results showed that the AG-3D ResNet model can segment the hippocampus without MRI using only CT scan images, which is comparable to the results obtained by physicians participating in the RTOG0933 phase II clinical trial. Zhao et al. (25) generated synthetic MRI through conformal atlas alignment as an intermediate step in hippocampal structure segmentation. Technological advancements have shown that this intermediate step can be bypassed, and hippocampal segmentation directly from CT scan images is comparable to manual contouring by physicians (24).

### MRI

**Figure 2:**
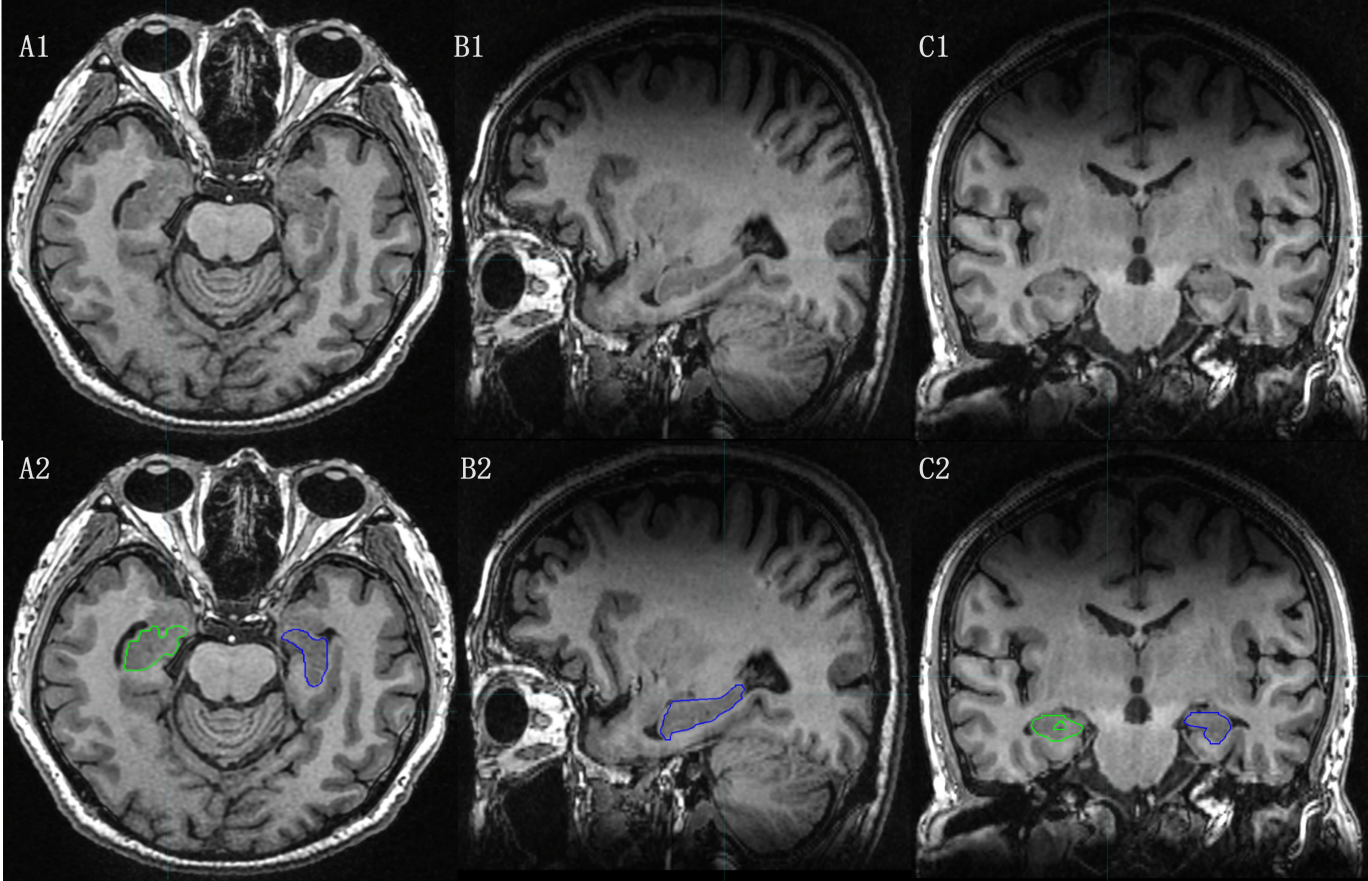
The hippocampal contour based on MRI T1-weighted Images. [**(A1)**, **(B1)**, **(C1)**:Axial, Sagital and Coronal images of MRI T1-weighted Images; **(A2)**, **(B2)**, **(C2)**: Axial, Sagital and Coronal images of MRI T1-weighted Images with hippocampal contour).

Compared to CT, MRI offers greater soft tissue resolution and plays a more significant role in diagnosing and distinguishing alzheimer’s disease (AD), mild cognitive injury (MCI), and assessing hippocampal structure (**Figure 2**).

The hippocampus is a two-layered gray matter structure with poorly defined borders on CT scans. According to the contour criteria of the RTOG0933 study protocol (26), the hippocampus was contoured on T1WI. The contouring process involved tracing a predominantly low-signal T1 area in the medial temporal horn, avoiding the anterior amygdala and hook gyrus, and locating the inner boundary at the lateral margin of the tetrapodal pool. The hippocampus is positioned posterior to the thalamus, with the temporal horn of the lateral ventricle remaining posteriorly. The contouring process stops at the appearance of the fornix peduncle on the posterior aspect of the tetrapodal pool, coinciding with the disappearance of the posterolateral low signal in the lateral ventricle.

Due to the limited image intensity contrast between the hippocampus and surrounding structures, various MRI contrasts can provide complementary information, and integrating multi-contrast images into modeling can enhance segmentation performance. Hu et al. (27) introduced an automated method for segmenting the hippocampus and amygdala in the presence of low background MRI contrast. This method combines level-set shape and active appearance modeling and utilizes multimodal data from T1WI, T2WI, and proton density-weighted MRI to improve segmentation. The results are promising. Ranjbar et al. (28) extracted texture and volume features of the hippocampus from brain MRI data to assess patients using the Clinical Dementia Rating (CDR) system. The goal was to differentiate between three groups of patients: those with normal cognition (CN), those with MCI, and those with AD. This study demonstrated the potential to identify early cognitive deficits using histological features of brain MRI.

Numerous studies have highlighted the importance of using MRI for hippocampal identification in computer-assisted diagnosis and neurological treatment (29, 30). Precisely determining hippocampal boundaries is essential for calculating volume and shape measurements. Research utilizing MRI to characterize hippocampal histological changes holds significant clinical promise.

## Automatic segmentation application for hippocampal contours

Chupin et al. (31) reported that segmentation of the hippocampus based on MRI may take up to 2hs and is a highly repetitive operation with high intra- and interoperator variability. To address these challenges and improve accuracy while reducing subjectivity and segmentation time, artificial intelligence (AI) and radiomic techniques have been integrated to facilitate automatic segmentation.

## Automatic and semi-automatic segmentation

Presently, two automatic contour methods are in use. One type relies on deep learning, particularly the convolutional neural network (CNN) algorithm, which trains on standard datasets and employs logical algorithms to generate accurate contours. The second method involves constructing a database of patient atlases using atlas libraries. Automatic segmentation is achieved by mapping these atlas templates onto new images using rigid or deformation alignment techniques.

## Deep learning and MRI-based hippocampal automatic segmentation

Deep learning techniques, particularly the U-Net segmentation framework based on CNNs, have made significant strides in medical image segmentation, especially for complex structures such as the hippocampus (32). Various automated methods for segmenting organs at risk (OAR) and tumor target areas have been developed based on this framework (33, 34).

Lin et al. (35) designed and evaluated a model for automatically segmenting the hippocampus using a pipeline of deep learning tools to generate scripts for VMAT RT treatment planning automatically for a fully automated treatment planning workflow. The overall mean dice similarity coefficient (DSC) and 95% hausdorff distance (HD) of the hippocampus were greater than 0.8 and less than 7 mm, respectively, and the HA-WBRT plan was automatically created in approximately 10 mins. An AI-assisted pipeline using deep learning tools allows rapid hippocampal segmentation and the generation of clinically acceptable HA-WBRT plans. Pan et al. (36) designed a variant network based on U-Net that consists of loc-Net and seg-Net segmentation models for automated hippocampal segmentation in WBRT. Loc-Net was used to determine the hippocampal location, while seg-Net accurately segmented the hippocampus. Their study reported mean DSCs of 0.86, 0.76, and 0.80 in the hippocampus for three different cohorts, along with mean average HDs of 1.8 mm, 3.1 mm, and 2.4 mm, respectively. Qiu et al. (37) explored the feasibility of using deep learning algorithms combined with MRI to segment the hippocampus automatically. Their research utilized two deep learning neural networks, Seg-Net and U-Net, for automated segmentation. The main deep neural network model can quickly retrain without requiring abundant data through transfer learning between the two neural nets. The mean DSC values for automatic and manual segmentation were 0.823 and 0.849, respectively. This level of precision, particularly for hippocampal structures approximately 3 cm^3^ in volume, is sufficient for clinical applications.

Automatic segmentation of the hippocampus using existing deep learning neural networks has achieved an accuracy close to that of manual contouring while significantly improving efficiency. This automated tool has the potential to assist in accessing hippocampal contours for BMs RT.

## Semi-automatic segmentation of the hippocampus based on atlas mapping

Although CNN-based hippocampal segmentation methods generally yield better results than traditional segmentation methods, the 3D U-NET neural network-based deep learning model structure involves multiple convolution and pooling processes, which can lead to information loss in small-volume OARs and introduce uncertainty in hippocampal volume contours (38). In contrast, the atlas-based atlas alignment technique effectively incorporates prior knowledge from existing atlases into the segmentation process, resulting in an accurate automatic segmentation technique.

Nobakht et al. (39) developed an atlas and CNN-based hippocampal segmentation tool called DeepHarp that generates standardized hippocampal segmentation. The Deep-Harp technique achieved an average DSC similarity of 0.88, surpassing other known hippocampal segmentation methods. Deep-Harp also demonstrated excellent reproducibility, with an average DSC of 0.95, outperforming published AI methods. The deep-Harp can automatically segment the hippocampus from T1WI datasets according to the ADNI-HarP protocol with high accuracy, facilitating atrophy measurements in various pathologies and contributing significantly to hippocampal volume measurements.

While some research has compared the geometric accuracy of automatically contouring OAR contours using both deep learning and atlas library methods, the general consensus is that the deep learning method yields slightly greater geometric accuracy than the atlas library method (40). Future research can focus on increasing the amount of data in the training set to incorporate additional features from tumor patients and further enhance the performance of the deep learning-based model, ultimately benefiting radiation oncologists and tumor patients alike.

## New RT techniques for hippocampal sparing

HA-WBRT aims to reduce the irradiation dose to the hippocampal region while ensuring sufficient irradiation to the entire brain. Advances in RT techniques have led to significant progress in HA-WBRT. **Table 3** Summary of hippocampal doses for HA-WBRT with selected RT techniques (41–45). The following are some typical modern techniques for hippocampal sparing:

**Table 3:** The dosimetric comparison of different techniques for HA-WBRT.

Study	Prescription dose	Radiation techniques	Dose constraints: mean dose to hippocampus	Comment
Xue et al. (2023) (41)	30Gy	C-VMAT NC-VMAT IMRT	12.20±0.54 Gy 11.71±0.48 Gy 12.18±0.33 Gy	NC-VMAT could significantly improve dose homogeneity and reduce the D50% in the brain.
Zhang et al. (2023) (42)	30Gy	Tomo VMAT	9.23±0.94 Gy 11.77±1.02 Gy	Compared with the VMAT plan of HA-WBRT, Tomo technology has significant dosimetric advantages
Takaoka et al. (2021 (43)	30Gy	Tomo Proton	11.1±0.50 Gy 7.0±0.26 Gy	IMPT achieved excellent hippocampus and scalp-sparing.
Canyilmaz et al. (2020) (44)	60Gy	C-VMAT IMRT	17.20 Gy 15.07 Gy	VMAT significantly reduced monitor units compared with standard IMRT and sparing IMRT
Aljabab et al. (2021) (45)	54Gy	C-VMAT Tomo Proton	17.20 Gy 15.90 Gy 14.70 Gy	IMPT has the strong potential to reduce the dose to the HPA and hippocampus

NC-VMAT, Non Coplanar-VMAT; C-VMAT, Coplanar- VMAT.

## Photon RT techniques

### Volumetric arc intensity-modulated RT

One of the advantages of intensity-modulated arc therapy (IMAT) or VMAT is the speed of irradiation delivery and optimal dose distribution.

**Figure 3:**
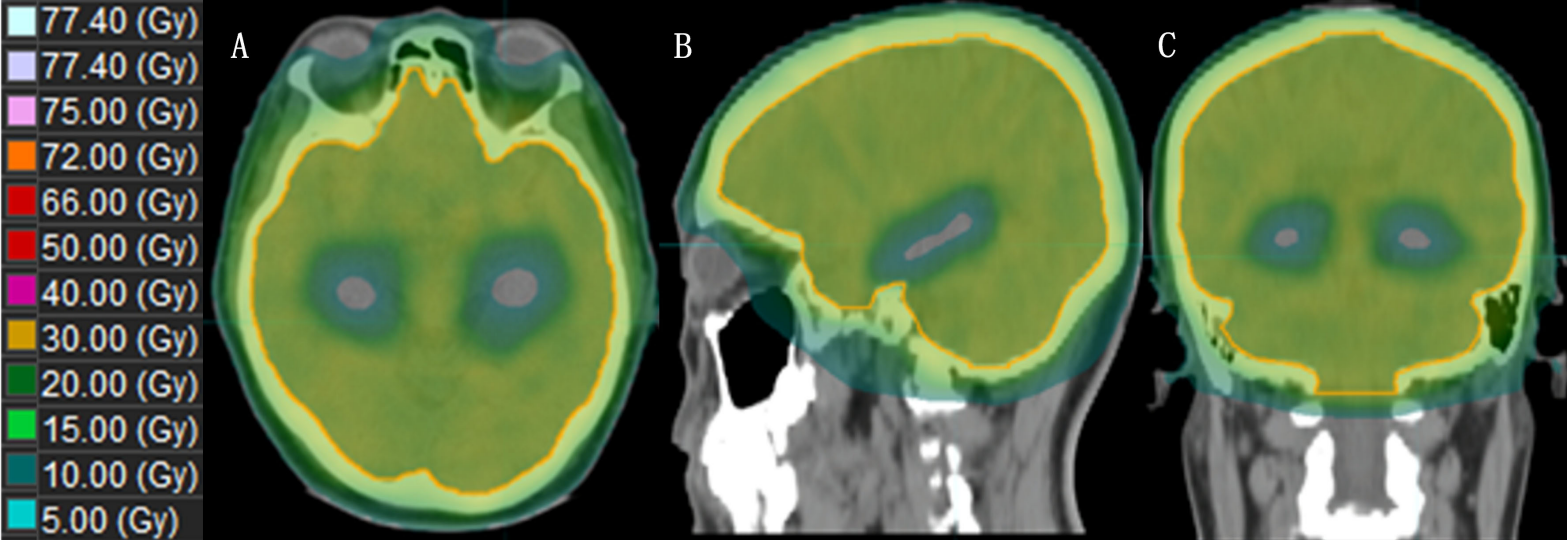
The dose distribution of HA-WBRT applying VMAT. [**(A–C)**: Dose distribution at the axial, sagittal and coronal views].

Xue et al. (41) demonstrated the dosimetric advantages of simplified Non-Coplanar VMAT (NC-VMAT) in HA-WBRT and compared it with those of IMRT and Coplanar VMAT (C-VMAT). It was found that after dose normalization (D95%=30 Gy), NC-VMAT could significantly improve dose uniformity, which decreased to D50% in the brain and D2% in the hippocampus. Li et al. (46) compared dosimetric differences between double-arc VMAT and 7-field IMRT (7F-IMRT) in patients with lung cancer BMs receiving HA-WBRT. They found that, compared with 7F-IMRT, double-arc VMAT provided superior coverage and homogeneity of the planned target area for hippocampal sparing. This approach also resulted in a lower mean maximal dose to the hippocampus and other areas, reducing the treatment time by 74%. Kim et al. (47) demonstrated that tilting the head at an angle of approximately 11° improved the dose distribution and reduced the irradiated dose in the hippocampus during HA-WBRT using the VMAT technique. Fu et al. (48) showed that the maximum and mean doses to the hippocampus from the modified VMAT regimen were 7.88 Gy and 6.32 Gy, respectively, which were significantly lower than those from the conventional VMAT regimen, and the maximum dose was lower than that from Tomo, with a significant dose advantage for tilted head positioning (**Figure 3**).

## Spiral Tomo

Tomo offers superior dose distribution compared to conventional IMRT, IMAT, and VMAT.

**Figure 4:**
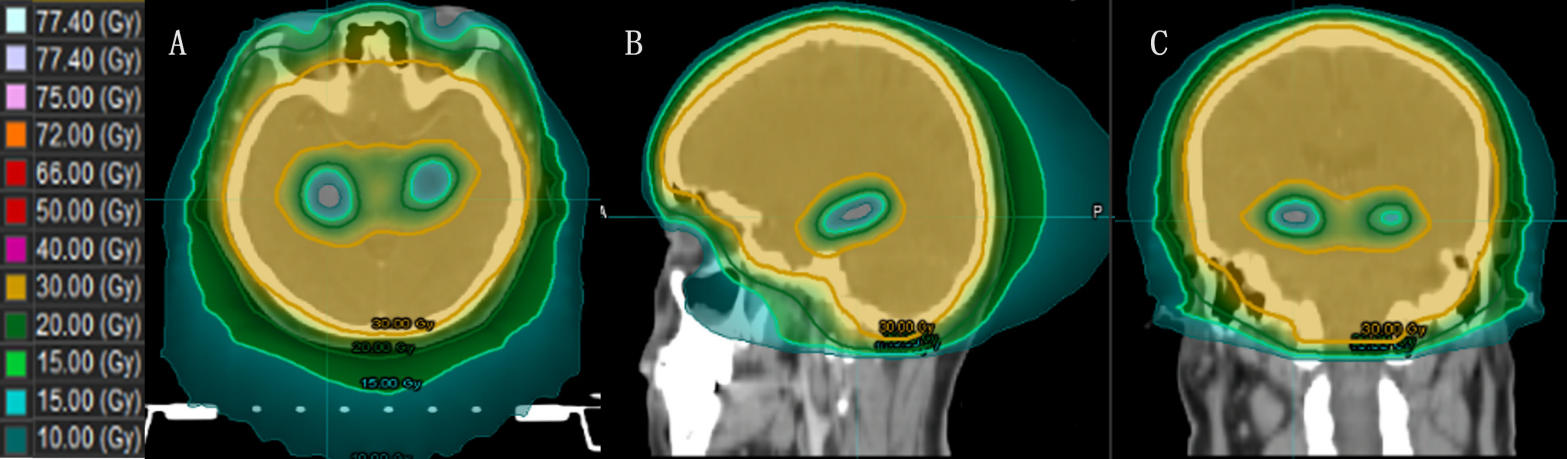
The dose distribution of HA-WBRT applying Tomo. [**(A–C)** Dose distribution at the axial, sagittal and coronal views].

Jiang et al. (49) compared the doses of four different treatment modalities for HA-WBRT: step-and-shoot IMRT (sIMRT), dynamic IMRT (dIMRT), VMAT, and Tomo. The Tomo plan had the lowest D100% to the hippocampus, making it advantageous for hippocampal sparing. Gondi et al. (17) reported that the median and maximum dose limits of Tomo for the hippocampus were reduced by 29.5% and 16.3%, respectively, compared to those of a conventional linear accelerator. Tomo’s faster dose fall-off and better hippocampal sparing have been proven, and HA-WBRT with Tomo has become the general treatment approach (**Figure 4**).

Tomo, on the other hand, may have overtreated the surrounding structures of the brain to minimize the dose to the hippocampus in the treatment of prophylactic cranial irradiation (PCI) and in pediatric patients, thereby increasing the risk of secondary malignancies. To assess this potential drawback of the Tomo scale, Marsh et al. (50) measured the overall dose of conventional IMRT and Tomo in uninvolved brain regions of patients with high-grade glioma (HGG). The results demonstrated an average 23% reduction in brain area dose after IMRT compared to Tomo across all treatment regimens tested. Despite the theoretical risk of localized overtreatment, the overall dose delivered by any technique is surprisingly low compared to that of a nonavoidance scheme. From this perspective, the use of conventional IMRT is considered the best method for protecting the hippocampus. Another method of reducing the overall dose to the brain is proton therapy.

## Proton RT techniques

The unique dose distribution pattern known as the Bragg peak in proton beam RT effectively reduces radiation exposure to normal brain tissue while maintaining treatment efficacy (51). This RT method holds promise for clinical application, particularly in hippocampal sparing.

In their study, Stoker et al. (52) included 10 pediatric and adult patients with neurological disorders and compared two RT techniques, IMPT and VMAT, in HA-WBRT, found that IMPT had a significant advantage in preserving the hippocampus, with the mean hippocampal dose decreasing from 13.7 Gy of VMAT to 5.4 Gy of IMPT for children and from 11.7 Gy to 4.4 Gy for adults. Notably, this study stands out for its inclusion of pediatric patients, who may exhibit greater sensitivity to radiation-induced cognitive deficits than adults. Research on the use of HA-IMPT for protecting cognitive abilities and enhancing quality of life in pediatric patients shows promise. Using the IMPT technique, Aljabab et al. (45) explored whether long-term neuroendocrine disruption and cognitive dysfunction resulting from craniofacial irradiation (CSI) could be mitigated by sparing the hippocampus and the hypothalamus-pituitary axis (HPA). The results indicated a decrease in the HPA-receiving dose from 32.2 Gy to 17.9 Gy and a reduction in the hippocampal dose from 39.8 Gy to 22.8 Gy. IMPT demonstrates strong potential for reducing both HPA and hippocampal doses while maintaining target coverage.

Florijn (53) and colleagues conducted a comparison of efficacy between IMPT and photon RT and revealed that IMPT achieved significantly greater Paddick conformity index (PCI) values and lower dose gradients (RXX%). To quantify the dose conformity, the PCI and dose gradients were derived. RXX% is the ratio of the volume receiving XX% of the prescribed dose to the volume of the clinical target volume (CTV). The advantage of hippocampal sparing with IMPT was also evident, as indicated by a reduction in the hippocampal Dmean from 4.6 Gy to 3.2 Gy, D40% from 3.5 Gy to 1.2 Gy, and Dmean in normal brain tissue from 5.7 Gy to 3.2 Gy, with a 22–47% reduction in volume receiving 10–30 Gy. Takaoka et al. (43) compared WBRT plans between Tomo and IMPT, highlighting IMPT’s superior target coverage over Tomo and its significant reduction in the dose received by the hippocampus compared to the scalp. Consequently, HA-WBRT using the IMPT technique proves to be an effective treatment option for preventing cognitive decline and hair loss.

## Limitations of this review

Due to the extensive literature on HA-WBRT, it is inevitable that some studies were not included in this review. We focused on studies closely aligned with our team’s future research. The papers cited in this review have now been covered to represent the current state of the art and related research.

## Future directions

**Figure 5:**
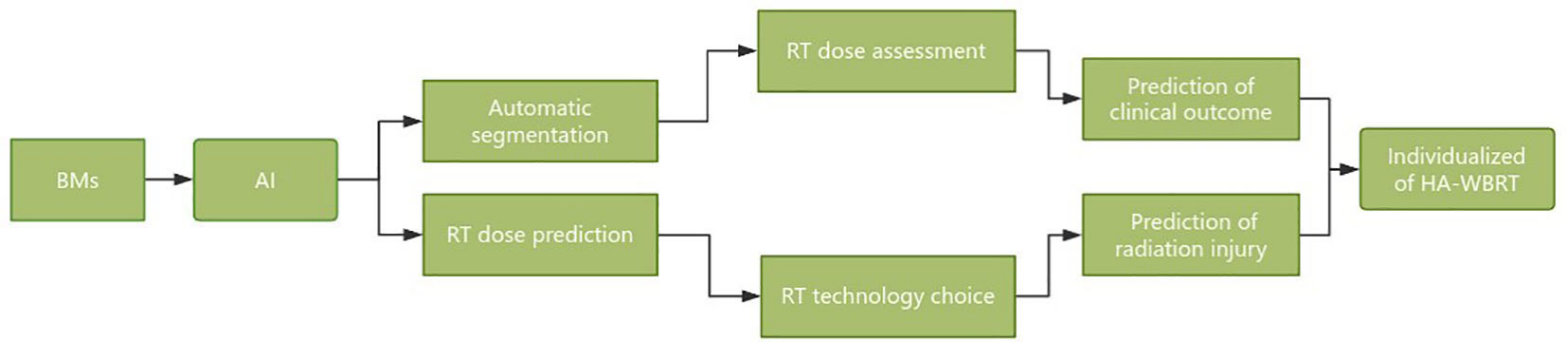
Flowchart of HA-WBRT development directions.

Hippocampal sparing is a critical concern that cannot be overlooked in WBRT and holds immense significance in enhancing patient prognosis and quality of life. The future development directions of HA-WBRT can be summarized as follows (**Figure 5**):

1. Automatic segmentation of the hippocampus and dose assessment based on AI technology.
2. Radiomics was applied to track microscopic changes in the hippocampus after WBRT and predict cognitive dysfunction.
3. Evaluation of the hippocampal RT response and radiation injury risk after WBRT based on multisequence functional MRI.
4. Automatic selection of HA-WBRT techniques and equipment based on dose prediction.
5. The WBRT dose was selected based on the risk of cognitive dysfunction and individualized protection of the hippocampus.
6. Integrated solution for HA-WBRT: automatic segmentation, dose prediction, efficacy, damage prediction, and follow-up tracking.

The utilization of AI segmentation technology in conjunction with advanced RT techniques substantially enhances the accuracy of hippocampal contouring. It reduces the hippocampal dose, ultimately enabling a greater number of patients with BMs to benefit from WBRT.

## Author contributions

RL: Conceptualization, Data curation, Writing – original draft, Writing – review & editing. GG: Formal analysis, Supervision, Writing – review & editing. KM: Conceptualization, Writing – review & editing. SD: Writing – review & editing. YY: Resources, Funding acquisition, Writing – review & editing.
